# Comparative analysis of grapevine whole-genome gene predictions, functional annotation, categorization and integration of the predicted gene sequences

**DOI:** 10.1186/1756-0500-5-213

**Published:** 2012-05-03

**Authors:** Jérôme Grimplet, John Van Hemert, Pablo Carbonell-Bejerano, José Díaz-Riquelme, Julie Dickerson, Anne Fennell, Mario Pezzotti, José M Martínez-Zapater

**Affiliations:** 1 Instituto de Ciencias de la Vid y del Vino (CSIC, Universidad de La Rioja, Gobierno de La Rioja), CCT, C/Madre de Dios 51, Logroño, España, 26006, Vietnam; 2 Bioinformatics and Computational Biology Department, Iowa State University, Ames, IA, 50011, USA; 3 Departamento de Genética Molecular de Plantas, Centro Nacional de Biotecnología, (CNB-CSIC), C/Darwin 3, Madrid, España, 28049, Vietnam; 4 Plant Science Department, South Dakota State University, Brookings, SD, 57007, USA; 5 Department of Biotechnology, University of Verona, Strada le Grazie 15, Verona, 37134, Italy

## Abstract

**Background:**

The first draft assembly and gene prediction of the grapevine genome (8X base coverage) was made available to the scientific community in 2007, and functional annotation was developed on this gene prediction. Since then additional Sanger sequences were added to the 8X sequences pool and a new version of the genomic sequence with superior base coverage (12X) was produced.

**Results:**

In order to more efficiently annotate the function of the genes predicted in the new assembly, it is important to build on as much of the previous work as possible, by transferring 8X annotation of the genome to the 12X version. The 8X and 12X assemblies and gene predictions of the grapevine genome were compared to answer the question, “Can we uniquely map 8X predicted genes to 12X predicted genes?” The results show that while the assemblies and gene structure predictions are too different to make a complete mapping between them, most genes (18,725) showed a one-to-one relationship between 8X predicted genes and the last version of 12X predicted genes. In addition, reshuffled genomic sequence structures appeared. These highlight regions of the genome where the gene predictions need to be taken with caution. Based on the new grapevine gene functional annotation and in-depth functional categorization, twenty eight new molecular networks have been created for VitisNet while the existing networks were updated.

**Conclusions:**

The outcomes of this study provide a functional annotation of the 12X genes, an update of VitisNet, the system of the grapevine molecular networks, and a new functional categorization of genes. Data are available at the VitisNet website (http://www.sdstate.edu/ps/research/vitis/pathways.cfm).

## Background

Due to its substantial economic importance and its position as a model species for perennial fruit crops [1,2], the *Vitis* genus, particularly the *Vitis vinifera* species, has benefited from a large effort to develop genomic tools and data [3-6]. Consequently, the bioinformatics resources for the grapevine species has expanded in the past few years, with a variety of tools created for post-genomics era applications [7]. Most notably, the genomes of the heterozygous variety Pinot Noir and a near homozygous Pinot Noir derived inbred (PN40024) have been sequenced [8,9]. The sequencing and the assembly of the latter have been updated recently from an 8X to a 12X coverage of the genome sequence and a 12X assembly. The 8X and 12X assemblies are accompanied by the respective gene structure predictions, which contain different types of subsequence predictions. These include genes, mRNAs, UTRs, introns, exons, and inter-genic spaces. The methods for gene prediction for the 8X genomic sequence were previously published [9], using the GAZE software [10]. Two versions of the 12X prediction are available. Version 0 (12Xv0) was performed with the GAZE software by the Genoscope in Evry, France. Version 1 (12Xv1) is the result of the union of v0 and a gene prediction performed with JIGSAW software [11] at the CRIBI in Padova, Italy [12].

The v0 prediction has been available online since 2009 on the NCBI website and the Genoscope website (http://www.cns.fr/vitis)*.* The v1 prediction, available at http://genomes.cribi.unipd.it/, was used to design the latest available gene expression microarray for grapevine, based on NimbleGen technology, which is the whole-genome array for grapevine. Other earlier microarray platforms have also been widely popular within the grapevine research community, in particular the two Affymetrix microarray platforms, the *Vitis vinifera* GeneChip® [13] based on the EST sequences assembled into contigs corresponding to the DFCI gene index version 4 (http://compbio.dfci.harvard.edu/tgi/cgi-bin/tgi/gimain.pl?gudb=grape) and the GrapeGen microarrays [14] based on the DFCI gene index version 5 complemented by the GrapeGen project EST sequences. Within this context, the assessment of the correspondence between all the different sets of grapevine genes (8X and 12X genome sequence coverage and EST) has several interests: (i) to provide correspondence tables to the grapevine scientific community that relate identical genes with different names, which would allow work performed with the 8X genome gene IDs to be updated to the 12X gene IDs; (ii) to use the functional annotation performed on the 8X predicted genes and to implement it easily on the 12X genome; (iii) to update VitisNet, which was based on the 8X prediction [15], for the 12X genome and therefore make it compatible with the NimbleGen array; and (iv) to allow comparison of results obtained from different high throughput platforms such as microarrays or proteomics studies.

## Results and discussion

### Sequence homology presents a cardinality problem

The two available gene predictions of the grapevine genome were produced from an 8X coverage assembly and from a 12X coverage assembly that included the genomic data from the 8X coverage version with Arachne [16]. GAZE software was used to perform the gene predictions for the 8X and the first version of the 12X (v0) prediction. The complete procedure is available in chapter 5 of the supplementary material in Jaillon et al. [9] and was modified to take into account whole transcriptome shotgun data from Solexa sequencing. The procedure for the 12Xv1 is described by Forcato [12]. The v1 gene prediction is the result of the integration between v0 and the CRIBI prediction that was performed with the JIGSAW software.

The comparison analysis performed by Megablast may result in some genes having a many-to-many relationship between 8X and 12X ORFs, with paralogous domains causing a confounding web of links between sets of genes. The degree to which a gene is linked to multiple sister genes in the other assembly versions corresponds to its cardinality with |gene 12X| = {gene 8X_1,_ gene 8X_n_} = n. To avoid incorrect matching, the chromosome position and matching results for adjacent genes were considered in addition to the sequence similarity.

It has been observed that the 8X prediction could define a single gene on a specific locus, while a slightly different assembly or prediction method in the 12X version defined several separate genes spanning the same nucleotides. This results in many 12X predicted genes aligning almost perfectly with the same 8X gene. In the case that multiple genes match different portions of a single gene, a possible solution to identify which is the correct prediction is to blast the genes against proteins from other species to observe (i) if the predicted protein either covers multiple similar proteins with different sequences on different regions of its sequence (considered artificial chimeras) or matches a single gene, or (ii) if multiple consecutive genes actually match a single protein or if each one matches a different single protein. Among the genes with cardinality issues, 2363 matched a protein with existence demonstrated at the protein level. After performing this validation, 147 genes from the 12Xv1 prediction seemed to have been wrongly assembled and needed to be split, resulting in a proposed 154 new genes (seven genes were triple chimeras). These genes were identified as “To split” in the Additional file 1 in the column “cardinality between 8X and 12X” and in Table 1. The new genes are identified with “_2” at the end of the v1 gene ID in Additional file 1. On the other hand, 1429 gene models seemed to have been incorrectly split in the 12X and needed to be reassembled. These genes were identified as “Overlap” in Additional file 1 and in Table 1. Also, 1774 genes were correctly merged in the 12X but split in the 8X assembly. These genes were identified as “merge” in Additional file 1. Finally, 428 genes were correctly split in 12X that had been incorrectly merged in the 8X assembly. These genes were identified as “Split” in Additional file 1 and Table 1. This last set of genes seems to contain a large proportion of genes that were not positioned on known chromosomes in the 8X assembly and have been placed on chromosomes in the 12X assembly.

**Table 1:** Number of predicted gene sequences from the 12Xv1 grapevine genome coverage showing cardinality values higher than one when compared with predicted genes in other versions and assemblies

	**Multiple 12Xv1 genes matching one gene in another set**			**Multiple genes in another set matching one 12Xv1 gene**		**Multiple situations**
	**Redundant**	**Overlap**	**Split**	**Merged**	**To split**	
Comparison 8X	623	1429	428	1774	147	122
Comparison mRNA	54	14	7			
Comparison 12Xv0	2735			846		5

Redundant: multiple genes match the same portion of one gene in other set. Overlap, Split, Merged, and To split: multiple genes match different portions of one gene in other set. Overlap and To split: 12Xv1 genes need to be modified respectively (either merged with another gene or split).

Discrimination of tandemly duplicated genes may be possible with the 12X coverage. A group of 623 12Xv1 predicted genes matched single genes in the 8X assembly on the same portion of their respective sequence. These genes were identified as “redundant” in Additional file 1 and Table 1. Some of these were consecutive 12Xv1 genes matching a single 8X gene, most likely corresponding to tandem repeat genes undetected in the 8X assembly. Another possibility is that two distinct bulks of consecutives genes match a single set of consecutive genes in the 8X, indicating potential mistakes in the 12X assembly. The possibility that multiple 8X genes match a single 12X gene has not been considered since it would most likely correspond to a situation in which the 8X gene prediction was incorrect. It is less likely that the 12X gene prediction was incorrect. There were 122 genes from the 12X assembly that showed a more complex matching pattern involving at least two of the conditions leading to a “many-to-many” relationship between genes. For example there were 61 cases where two 12X annotated genes (*A* and *B*) aligned on different regions of one 8X gene (*C*) but another portion of one of them (*B*) matched to a second 8X gene (*D*). As a result, 3 correct answers were possible: keeping gene *A* and gene *B* separated (no change); or a portion of gene *B* needed to be assembled with gene *A*; or gene *A* and gene *B* needed to be merged. BLAST analysis was also performed on the non-redundant set of grape mRNAs from the DFCI (Grape gene index v5), resulting in fourteen 12X genes that needed to be merged. For these genes, the comment in Additional file 1 in the cardinality between 8X and 12X column is followed by “mRNA”.

Similar matching between 12Xv0 and 12Xv1 assembly versions was also performed. This identified 3581 genes with potential mistakes in structural annotation, thus presenting a degree of cardinality higher than one in 12Xv1. Among these, 1187 genes were identified in the comparison with the 8X.

**Figure 1:**
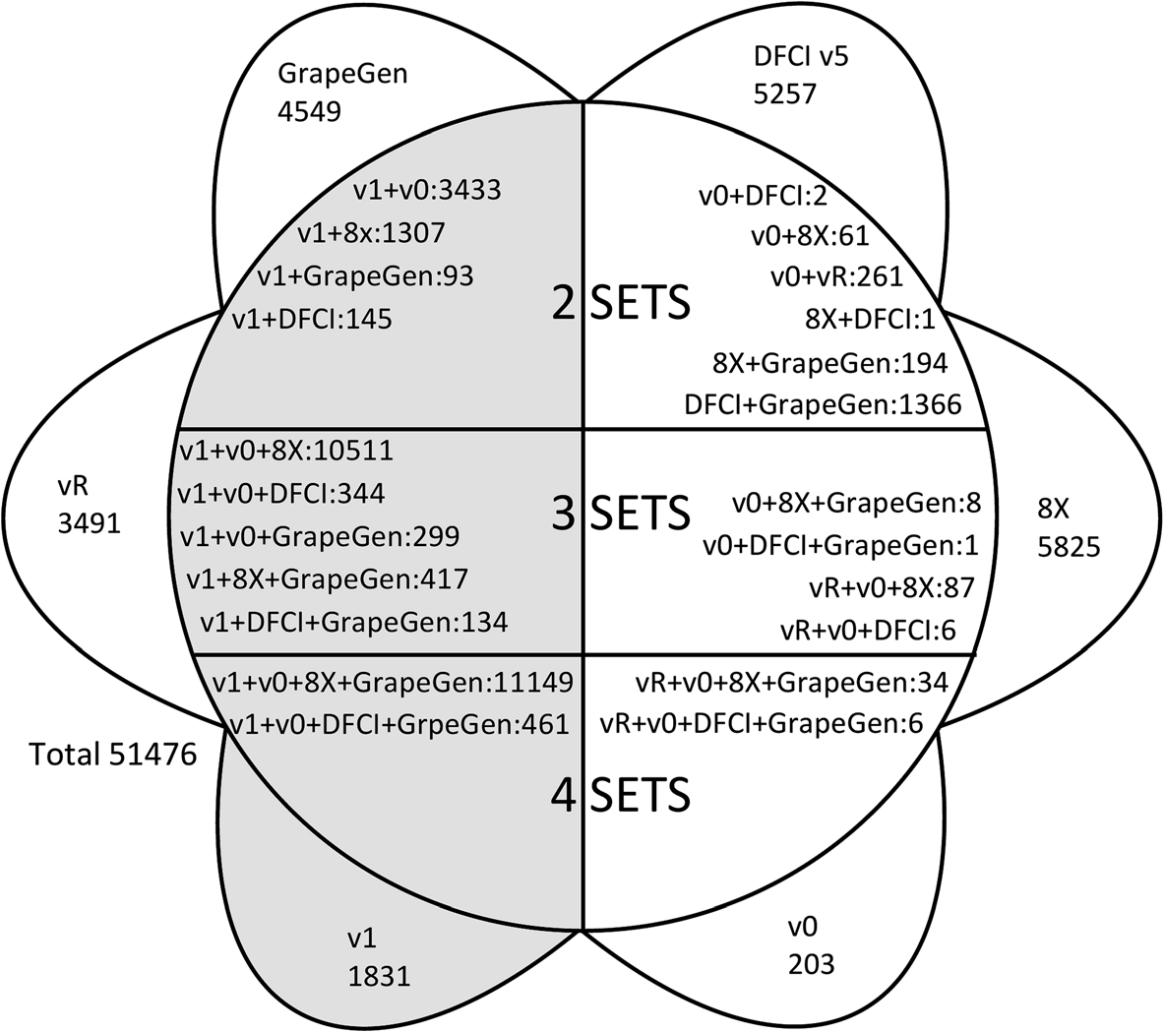
**Representation of overlap between the different sets of predicted gene sequences (51476) available for grapevine.** 8X: genes identified in the 8X coverage genome sequence; DFCI v5: mRNA sequences identified in the DFCI gene index EST sequence repository version 5; v1: genes identified in the 12X coverage genome assembly, version 1 of the gene prediction; V0: genes identified in the 12X coverage genome assembly, version 0 of the gene prediction; VR: predicted genes from the repeat track of the 12X coverage genome sequence, version 1 of the gene prediction; GrapeGen: mRNA sequences identified in the set of mRNA used to construct the GrapeGen Affymetrix microarray; Grey: genes present in the latest update of the protein prediction (12Xv1).

Most genes did not show cardinality >1 and a one-to-one relationship between 12Xv1 and 8X could be established for 18,725 genes (Additional file 1). However, 6740 predicted genes from the 12Xv1 assembly were not found in the 8X assembly gene prediction, and among them 1342 predicted genes matched transcripts in the EST database (Figure 1). In contrast, 6020 predicted genes in the 8X gene prediction were not found in the 12X gene prediction (69 additional genes where only found in the v0). In addition, there were still 11172 non redundant EST sequences from grapevine that did not match any gene from all the public sequenced and assembled grapevine genomes: 5257 from the DFCI v5, 4549 included in the GrapeGen Affymetrix microarray and 1366 from both. Since the 12Xv0 prediction was considered during the construction of the 12Xv1, there were only 275 genes from v0 that were not retrieved in v1. They most likely correspond to sequences of low quality that have been filtered out. Additionally, the 3885 genes of the repeat track from the 12Xv1 were not considered for matching with the 8X gene prediction although 394 of them matched 12Xv0 sequences. The repeat track contains highly speculative filtered out (masked) genes, so it was included in this analysis with caution because few of those sequences may be actual genes. The difference between the compositions of the predicted gene sets may be explained by multiple causes. It is reasonable to assume that the 12X gene prediction is better since it included a higher coverage and an updated gene prediction method from the one used for the 8X assembly. Therefore, detection of new genes in the 12X was expected. The absence of almost as many genes from the 8X prediction is more surprising. It may be related to a better detection of false positives, but there is a possibility that these genes were wrongly undetected in the 12X gene prediction. For that reason they were kept in the set of unique genes for future analyses.

### Relative position of predicted gene sequences

**Figure 2:**
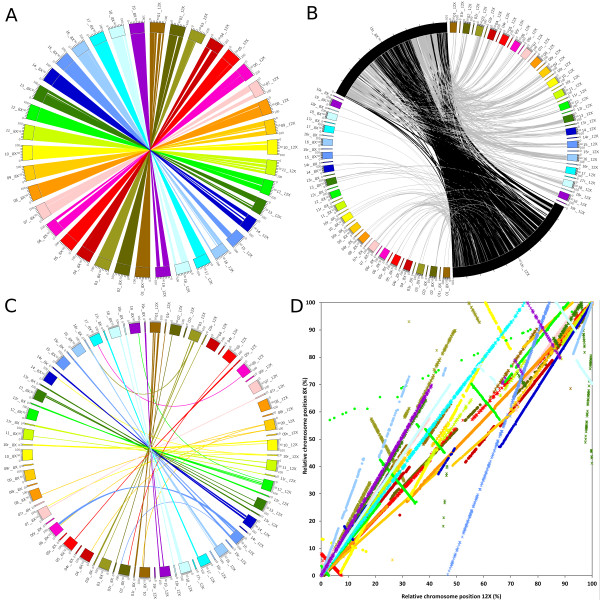
**Plots of the relative position of predicted genes between the 12Xv1 and the 8X coverage assemblies of the grapevine genome sequence.** Color code representing chromosomes and genes is identical in the 4 images. Axes represent percentage of the total length of each chromosome. Labels indicate chromosome and corresponding assembly. A) Relative position of genes on the same chromosome number in both assemblies. Colors of the links are identical to the 12X chromosome of origin. B) Relative position of genes in the unknown chromosome in at least one assembly. Small chromosomes marked with “r” represent random chromosomes and were arbitrarily set to 1/10th the regular chromosome size. Unknown chromosomes are magnified 20X relative to regular chromosomes. Black links represent genes belonging to the unknown chromosome in both assemblies. Grey links represent genes belonging to the unknown chromosome in only one assembly. C) Relative position of genes in two different chromosomes in the two assemblies. Small chromosomes marked with “r” represent random chromosomes and were arbitrarily set to 1/10th the regular chromosome size. Colors of the links are identical to the 12X chromosome of origin. D) Relative position of genes on the same chromosome number in both assemblies. To avoid confusion within similar colors, stars represent genes from a odd numbered chromosome and circles represent genes from a chromosome with an even number.

The relative position of predicted genes in the 8X and the 12Xv1 genome coverage sequences was compared as shown in Figure 2[2]. This figure plots the 23188 unique sister gene pairs. Figures 2A and 2D show the 16997 genes belonging to the same chromosomes in both assemblies. Figure 2B shows the 4384 genes that were attributed to the unknown chromosome in at least one assembly. Figure 2C shows the 1807 genes that were allocated onto 2 different chromosomes or were not positioned in a chromosome (random chromosome) between the two assemblies. Sister pairs were coded with colors specific to each chromosome and either stars (odd numbered chromosomes) or circles (even numbered chromosomes) in Figure 2D. Many pairs present a pattern close to x = y with only slight deviation (Figure 2D). This shows that sister genes were detected in agreement between both genome versions.

However, important changes between the 8X and the 12X assemblies are apparent. These changes can be grouped into four modification types:

i. Genes with unknown chromosomal location in the 8X that were successfully placed on existing chromosomes in the 12X (Figure 2B, grey link, 3177 genes). Most of these genes were located inside the first or the last third of the 8X unknown chromosome sequence. The middle third of the unknown chromosome contains genes that were assigned in the unknown chromosome both in the 8X and the 12X assemblies (black links). The middle region contains smaller scaffolds which represents a problem, since increased sequence coverage had no effect on their assignment within chromosomes. In the 8X assembly unassigned scaffolds were aggregated on the unknown chromosome more or less by size, with the largest towards the edges and the smallest towards the center. It is not surprising that small scaffolds could not originally be assigned to a chromosome as their short length decreases the probability that they will encounter markers linking them to the genetic maps; however, after the 12X assembly they still were not merged with other scaffolds or their scaffold size did not increase. It is possible that these scaffolds belong to regions difficult to sequence, such as heterochromatin. It has been shown that in *Anopheles gambiae,* heterochromatin is widely present in unknown chromosome [17]. Syntenic approaches have been used successfully in dog (with human) to clear the ambiguity of unknown chromosome scaffolds, but it is only applicable to scaffolds containing at least 3 genes [18].

ii. In the 8X assembly some predicted genes were assigned to a given chromosome but were not positioned within it. A significant portion of those predicted genes were definitively located on a chromosomal position (Figure 2C, 1570 genes) in the 12Xv1 assembly. The most notable sets of newly positioned genes were on chromosomes 1, 15 and 18, with more than 200 genes positioned for each chromosome.

iii. In the 12X assembly whole chromosome sections were inverted without changing their location (Figure 2D, 1444 genes with a pattern describing a negative correlation between x and y). This is particularly relevant on chromosomes 3 (khaki star, 230 genes), 5 (red star, 174 genes), 7 (pink star, 46 genes) 10 (yellow circle, 45 genes), 11 (light green star, 26 genes) 12 (green circle, 518 genes), 13 (dark green star, 53 genes), 14 (blue circle, 86 genes), 18 (light turquoise circle, 187 genes) and 19 (purple circle, 79 genes). Large assembly inversions are most likely caused by sequencing errors on the inversion flanks or ends. These errors could be the result of low coverage in those regions.

iv. One hundred one predicted genes changed chromosomal assignment (Figure 2C). These can be identified by the links describing curves in the figure. A large portion (70 genes) of the 8X assembly chromosome 6 was placed on chromosome 15 of the 12X assembly. A smaller group (17 genes) from the random chromosome 16 was definitively attributed to chromosome 3. An additional 14 genes were assigned to different chromosomes in the two assemblies.

Overall the 12X assembly shows a clear increase in its accuracy regarding scaffold assignment since a substantial portion of the 8X unknown chromosome and unidentified flanking areas were positioned in the 12X assembly. Figure 2A visualizes the regions that were modified between the assemblies. More specifically, the missing parts in the 12X chromosomes corresponded to transferred parts visible in Figures 2C and 2D. The size of the unknown chromosome dropped from 150 Mb in the 8X assembly to 45 Mb in the 12X assembly. Relatively few regions have been wrongly assigned to a chromosome in the 8X. Since the 12X assembly is assumed to be more accurate than the 8X, and few wrongly assigned regions were found between assemblies, the degree of confidence in the scaffold assembly of the 12X coverage version should be rather high. However, it should be noted that the direction of the assembled scaffold seems to present a high level of discrepancy between the two assemblies, so it is reasonable to assume that accuracy in the 12X assembly is still not quite optimal.

### Functional assignment

**Figure 3:**
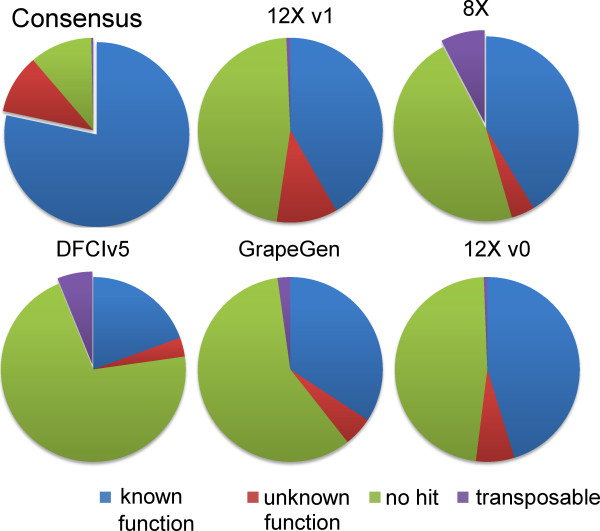
**Classification of predicted genes in the consensus set of sequences and the sets of orphan sequences based on their sequence matching results.** Blue: predicted genes matching sequences that have an assigned molecular function. Red: predicted genes matching sequences that lack assigned molecular function. Green: predicted genes not matching any known sequence from other species. Purple: predicted genes matching genes from other species considered as viral, transposable elements or related sequences. Consensus: genes of the 12Xv1 shared with another set. 12Xv1: genes unique to the 12X coverage genome sequencing version 1 of the gene prediction. 8X: genes unique to the 8X coverage genome sequencing. DFCIv5: mRNA sequences unique to the DFCI gene index v5. GrapeGen: mRNA sequences unique to mRNA sequences used to construct the GrapeGen Affymetrix microarrays. 12Xv0: genes unique to the 12Xv0.

The consensus set of all unique sequences and the sequences unique to any subset were independently compared to sequences from other species and categorized into four main groups (Figure 3): sequences matching another sequence with (i) a known function, (ii) an unknown function, (iii) viral and transposable elements or related sequences and (iv) sequences that do not match any other sequence in public protein databases. Three quarters (75%) of the entire set of 12Xv1 genes could be categorized in the first three groups while approximately 15% did not match anything. In the five other predicted gene subsets, where predictions were only found in one group, the value for group (iv) reached at least 45%. This situation is likely caused by the existence of very short sequences and also explains the difficulties in matching them to the 12X assembly. In addition, viral and transposable elements represent a relatively high percentage (>5%) of the specific predicted genes within the EST and the 8X assembly gene prediction that were not retrieved in the 12X assembly. These genes were filtered from the 12Xv1 gene prediction, although the repeat track contained a significant proportion of viral sequences. The total consensus set of sequences corresponded to non-redundant sequences in the core set of genes that were already functionally annotated. A majority of these sequences not present in the 12Xv1 may correspond to untranslated or already existing sequences. However, uncertainty remains as to which of these are existing genes but undetected because of short sequence lengths. Match discrepancies in ESTs (DFCI grape gene index v5 and GrapeGen) could also be related in part to varietal specificities.

### Functional categorization

The total 51,476 predicted genes that are potentially unique were assigned to functional categories. For gene categorization, a plant physiology-oriented catalogue was constructed. The catalogue is based on MIPS functional categories, but is complemented by using terms from the GO catalogue that have been hierarchized and fitted within the MIPS categories to add a higher level of detail. For example, the MIPS subcategory for heavy metal ion transport lacks resolution. Therefore, subcategories corresponding to GO terms for aluminum, zinc, iron and copper transport were added. Additional attribution of functional categories was performed by converting the VitisNet networks, which frequently corresponded to existing categories, into functional categories. Genes present in a network were considered to belong to the corresponding category. However, transcription factor families in VitisNet corresponding to categories from the transcription factor databases planttfdb [19] or plntfdb [20] are new with regard to MIPS and GO categories. New categories were created and most corresponded to families of genes related to specific plant pathways, particularly those associated with secondary metabolism. The full list of categories and their correspondence with GO and MIPS categories is in Additional file 2. This correspondence allows translation of categories to GO or MIPS. There are 1595 functional categories that contain up to 8 levels of detail. The 12X predicted genes were allocated to a total of 970 categories. Given the limited experimental information available, most of the unused categories for grapevine genes corresponded to localization (cellular, cell type, tissue and organ).

**Figure 4:**
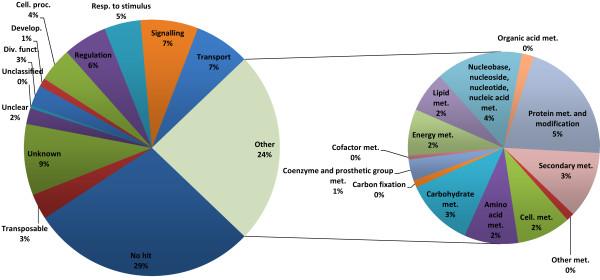
**Functional category distribution of the total non-redundant set of predicted genes.** The left pie chart equals higher level categories. The right pie chart equals secondary level categories within the metabolism category.

Figure 4 shows the repartition of the total non-redundant set of predicted genes into higher level functional categories in the primary chart on the left and the repartition within the metabolism category in the secondary chart on the right. The metabolism category is slightly overrepresented when compared to global category values in the literature (closer to 20%) because a choice was made to include nucleic acid metabolism here instead of placing it into cell fate and DNA processing. Almost half of the genes (44%) belong to poorly informative categories such as no hit, viral and transposable element, unknown (limited information about the function, presence of a known motif, or known to bind a molecule), unclear (the corresponding protein is somewhat known, but its exact role cannot be determined), or unclassified (the function is not related to any category). Predicted genes belonging to these categories cannot be assigned to lower level categories and would be difficult to place on the VitisNet network without further evidence of their molecular function. One fifth of all the genes (10,008) were present in two or more distinct categories.

### VitisNet network update

VitisNet is a bioinformatics tool that allows the simultaneous integration of “omics” data within grapevine molecular networks [15]. Hence it provides a fast and easy way to monitor the changes in abundance of molecules during a given experiment. Based on the new grapevine genome annotation and the functional sub-categorization of predicted genes, twenty eight new molecular networks have been created for VitisNet. These networks correspond to newly created networks from the KEGG website as well as the transcription factor families recently created on the plant transcription factor databases planttfdb [19] or plntfdb [20]. Among the new networks, two are related to metabolic pathways: glucosinolate biosynthesis and ABA biosynthesis. Seven networks are related to genetic information processing: RNA degradation, spliceosome, ribosome biogenesis in eukaryotes, RNA transport, mRNA surveillance pathway, sulfur relay system, and protein processing in endoplasmic reticulum. Two new networks are related to environmental information processing: plant-pathogen interaction and the R proteins from plant-pathogen interaction. Two networks are related to cellular processes: peroxisome and phagosome. Finally, fifteen transcription factors families were added: COAP15, GNAT, IWS1, MED, MTERF, OFP, KIP1-SANTA, WRC, nozzle, RCD1, SOH1, SWIB, TRAF, SWI-SNF-SWI3, PAH. Existing networks were also updated with the addition of the predicted genes that are specific to the 12X as well as genes for which annotation has been revisited. To date, 16,364 (68%) of the 24155 well characterized genes (belonging to categories other than no hit, transposable element, unclassified, unclear, and unknown) are localized on the molecular networks. The complete list of the networks and numbers of genes, proteins, and metabolites within them are available in Additional file 3.

## Conclusions

The main advantage of developing a unified nomenclature is the ability to port works performed on previous versions of grapevine sequencing projects (both genome and EST). Albeit valuable sources of information, multiplication of grapevine genome sequence sets creates a web of confusion in the analysis of the grapevine genome. The independent development of successive assemblies and gene annotation also results in a loss of information due to the absence of crosschecking between different annotation sources. By studying the differences between the 12X assembly gene prediction and other gene sets, the work reported here highlights significant discrepancies between the gene predictions from the two genome assemblies. In addition, a significant improvement in the manual functional annotation and categorization of the predicted genes was developed.

## Methods

### Predicted genome sequences alignments

Predicted grapevine sequences corresponding to probable mRNAs were compared in two ways with Megablast [21] between the 8X and 12X coverage sets of putative sequences. First, the predicted sequences from the 8X coverage were the query and the 12Xv1 predicted sequences were the subject database. Second, the predicted sequences from the 12Xv1 were the query and the 8X predicted sequences were the subject database. Default Megablast parameters were modified to compare sequences with high stringency, considering only a percentage of identity higher than 95% and an e-value lower than 1e-20, and displaying only the four best matches. The two BLAST outputs were manually inspected to define the potential true identical sequences according to the results of neighbor genes: at least two consecutively positioned genes from the 12X version had to hit two consecutive genes in the 8X. Since chromosome sequences have been largely revised, gene positions according to the absolute value within chromosome could not be accurately used to assess the identity between two sequences. Consequently, the relative position between genes was preferentially used.

Cardinality-related discrepancies between the two assemblies identified two groups of genes that required examination (i.e. genes from one assembly presenting a one-to-many relationship with genes from the other assembly). The first group included genes matching multiple counterparts of the other assembly on the same region of their sequence. These might be either tandemly repeated sequences misidentified only once or wrongly duplicated in one assembly, or redundant portions previously assigned to the unknown chromosome. The second group included genes with different regions of their sequence hitting multiple counterparts of the other assembly. The 12X genes belonging to this group (the one 12X hitting many 8X and the many 12X hitting one 8X) were blasted against public protein sequences (Uniprot) to check if two or more sequences corresponded to parts of at least one similar gene from other species. If positive, they were considered unique sequences that needed to be merged. If a sequence hit multiple proteins with different sequences from other species, it was considered as a potential chimera and needed to be split (see the workflow in Additional file 4). Since this step can only be done on one assembly, the genes from the 12X were preferentially chosen to validate the functional annotation. However, for doubtful situations, the 8x genes were compared to public databases.

Comparison between v1 and v0 annotations of the 12X sequence was performed with the same Megablast parameters. However, since the global chromosome sequence structures are identical, gene sequence positions on chromosomes must overlap. Cardinality was also established between the 12Xv1 and the 12Xv0 but no check was conducted using other species’ proteins as was done in the 8X to 12Xv1 comparison.

For the comparison of the 12Xv1 predicted genes with grapevine transcript sequences, hits were considered when homology was higher than 95% on a length longer than 100 bp and with an e-value ≪ e-20. The 100 bp span criterion was used to compensate for the inability to compare with neighbor genes, as is done in genomic sequence to genomic sequence comparisons. Transcript sequences corresponded on one side to the DFCI Grape gene index v5 and on the other side to the GrapeGen project sequences [22]. EST sequences in these sets are primarily from the cultivars Cabernet Sauvignon (half of the EST sequences), Chardonnay, Thompson Seedless, Muscat Hamburg, and Perlette.

### Gene annotation

The total set of genes was built from all genes of the unmodified 12Xv1 prediction complemented with the genes from the other transcript sets and gene predictions that were not identified in 12Xv1. For each gene, the sequence used for comparison with other species and the unique ID was defined according to the following priority order between sets: 12X sequencing v1 > 12X sequencing v0 > 8X sequencing > EST from the DFCI Grape gene index v5 > EST from the GrapeGen project.

Functional annotation was performed, when possible, by inferring the functional annotation of the 8X genes previously defined [15] to the new set of genes. A *de-novo* functional characterization was conducted as previously described [15] for genes that were absent from the previous unique gene set. Functional annotation of genes suspected to be involved in newly constructed molecular networks (since the previous release of VitisNet) was re-analyzed even when genes were found in the 8X prediction. In addition, all the genes presenting cardinality issues were functionally characterized independently of the function assigned in the 8X set.

### VitisNet networks construction

New networks were constructed since the last update of VitisNet. The new networks were constructed following the protocol described for each network class [15]. The transcription factor networks were completely re-designed since the layout of the transcription factors depends on the phylogenetic relationship of all sequences. Finally, all the 8X-type gene names were replaced by the 12X-type gene IDs according to the latest nomenclature.

### Functional categorization

A functional categories catalog was built by merging categories from the MIPS functional catalog [23] existing in plant species with a selection of GO terms [24] related to plant biological processes and categories related to the VitisNet networks, the transcription factors categories and the TCDB categories [25]. Categories were manually attributed to the genes according to their predicted molecular or physiological function.

## Competing interests

The authors declare that they have no competing interests.

## Authors’ contributions

JG designed the study, performed the analysis, and drafted the manuscript. JVH and MP participated in the design of the comparison between the 8X and 12X genome sequences. PCB, JDR and JMMZ participated in the functional assignment and categorization. AF participated in the conception and the update of VitisNet. All authors reviewed the manuscript. All authors read and approved the final manuscript.

## Supplementary Material

Additional file 1 **The complete grape gene annotation and correspondence between the sets of sequences.*Unique ID:*** ID from the highest priority level available for a unique gene sequence (priority order 12X sequence v1 > 12X sequence v0 > 8X sequence > EST from DFCI Grape gene index v5 > EST from Grapegen microarrays); gene name followed by an underscore and a number are theoretic genes corresponding to new genes that were incorrectly merged. ***Old 12Xv1 name:*** former name utilized for the v1 of the 12X sequence. ***12Xv0 ID:*** ID from the v0 of the 12X assembly. ***Identical genes in 8X or other EST:*** ID of the corresponding gene from the 8X sequencing or EST sequence that does not match an 8X gene. ***Probeset grapegen:*** probeset ID for the Affymetrix GrapeGen *Vitis vinifera* Genome Array. ***Chromosome position 12X:*** position of the gene on chromosome in the 12X sequencing assembly; the first part separated by underscore corresponds to the chromosome number, the middle part to the beginning position, and the last part to the end position. ***Cardinality between 12Xv0 and 12Xv1:*** Comment about the accuracy of the gene prediction inferred from the v0 to v1 comparison; “merge” indicates that multiple sequences of the v0 match one sequence of the v1, “partial” indicates that multiple sequences of the v1 match one sequence of the v0, numbers indicate how may genes from one set match one gene from the other set. ***Cardinality between 8X and 12Xv1:*** Comment about the accuracy of the gene prediction inferred from the 8X to 12X comparison; “merge” indicates that multiple sequences of the 8X assembly match one sequence of the 12Xv1 (unless noted otherwise the 12X assembly gene is correct), “To split” indicates that the 12X gene is incorrect and needs to be split (if there are more than 2 genes, those that need to be grouped are indicated by order in the column “Identical genes in 8X or other EST”), “redundant” indicates multiple12X genes matching a single 8X gene on the same position, XX indicates no match between 12X and 8X, OK indicates a one-to-one relationship between 12X and 8X, “OK (Split)” indicates a 12X gene matching an 8X gene that was an incorrect merging of multiple genes, “Ls” indicates a low score between the matches even though they seem to be correct. ***Track 12Xv1:*** the track of the 12Xv1 assembly, either the main track (v1) or the repeat track (v1_r). ***Functional annotation:*** tentative functional annotation; briefname, EC or Kegg ID: the identifier that is used in the networks. ***Network:*** list of the VitisNet networks in which the gene appears. ***Functional category:*** each functional category assigned to the gene. There are up to seven categories for a single gene. ***Best Arabidopsis match:*** best matched hit in *Arabidopsis* putative proteins. ***Gene Ontology (GO):*** list of the identified GO terms and their description. ***Plant Ontology (PO):*** list of the identified PO terms and their description. ***Pfam:*** list of the domains detected from Pfam. ***Smart:*** list of the domains detected from Smart. ***Prosite:*** list of the domains detected from Prosite. ***Psort:*** list of the cellular localization detected from Psort. ***InterPro domain:*** list of the domains detected from Interpro. ***Accession UniProt for published grapevine protein:*** UniProt ID for grapevine proteins individually published apart from the genome sequencing. ***Chromosome position 8X:*** position of the gene on chromosome in the 8X sequencing assembly. ***Other Vitis:*** presence in non-vinifera *Vitis* species. ***cDNA array***: ID used in the cDNA array from Mathiason et al. (2009). ***TC from VVGI5:*** list of other TC from the DFCI matching the gene. ***GeneChip probesets:*** probeset ID for the Affymetrix GeneChip® *Vitis vinifera* (Grape) Genome Array. ***Best match against proteins with evidence at protein level:*** compared with uniprot database. Click here for file

Additional file 2 **List of the*Vitis*putative proteins’ functional categories and correspondence with other catalogs.*Vitis Functional Category:*** full name of each functional category. ***Vitis Functional Category Code:*** numbered nomenclature of the *Vitis* Functional Category. ***Level:*** hierarchized level of description of the functional category. ***GO Name:*** full name of each GO description. ***GO ID:*** numbered nomenclature of the GO. ***VitisNet Network:*** Corresponding VitisNet network. ***MIPS Funcat Name:*** full name of each MIPS functional categories. ***MIPS Funcat:*** numbered nomenclature of the MIPS functional category. ***Number of genes:*** number of genes belonging to the category. Click here for file

Additional file 3 **List of networks available in VitisNet.*VVID***: VitisNet identification number; ***gen***: number of genes in network; ***pro***: number of proteins in network; ***met***: number of metabolites in network. New networks are italicized. Click here for file

Additional file 4 **Analyses workflow for determining cardinality between 8X and 12Xv1 assembly genes.** Straight line: representation of the genes from the 12Xv1 assembly. Wavy line: representation of the genes from the 8X assembly. Dotted line: genetic sequence. Click here for file
