# Supplementary figures and images for: Comparative analysis of grapevine whole-genome gene predictions, functional annotation, categorization and integration of the predicted gene sequences
Source: BMC Res Notes. 2012 May 3;5:213. doi: 10.1186/1756-0500-5-213 (PMC3419625; doi:10.1186/1756-0500-5-213)

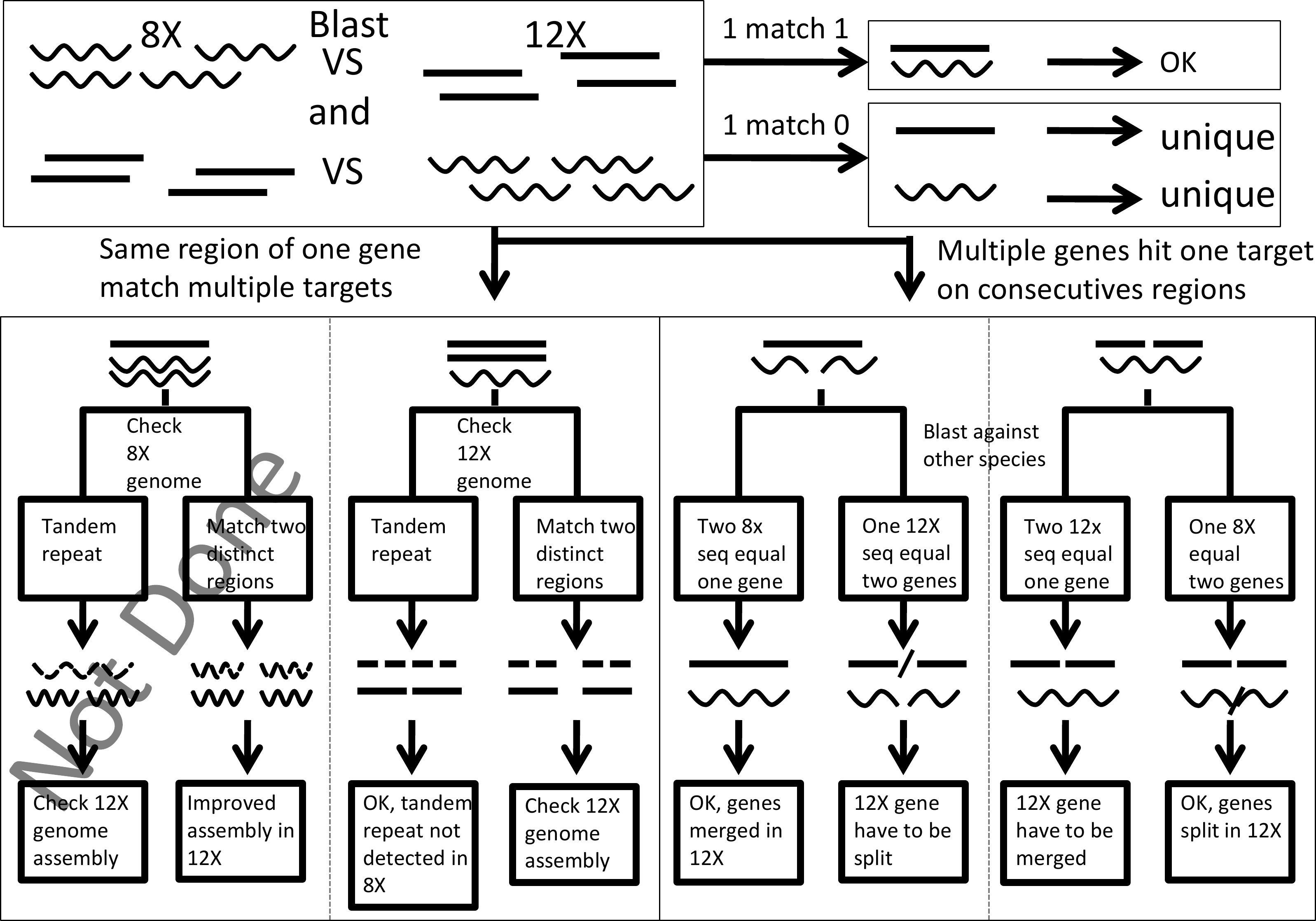

Supplement: Additional file 1 — The complete grape gene annotation and correspondence between the sets of sequences.Unique ID: ID from the highest priority level available for a unique gene sequence (priority order 12X sequence v1 > 12X sequence v0 > 8X sequence > EST from DFCI Grape gene index v5 > EST from Grapegen microarrays); gene name followed by an underscore and a number are theoretic genes corresponding to new genes that were incorrectly merged. Old 12Xv1 name: former name utilized for the v1 of the 12X sequence. 12Xv0 ID: ID from the v0 of the 12X assembly. Identical genes in 8X or other EST: ID of the corresponding gene from the 8X sequencing or EST sequence that does not match an 8X gene. Probeset grapegen: probeset ID for the Affymetrix GrapeGen Vitis vinifera Genome Array. Chromosome position 12X: position of the gene on chromosome in the 12X sequencing assembly; the first part separated by underscore corresponds to the chromosome number, the middle part to the beginning position, and the last part to the end position. Cardinality between 12Xv0 and 12Xv1: Comment about the accuracy of the gene prediction inferred from the v0 to v1 comparison; “merge” indicates that multiple sequences of the v0 match one sequence of the v1, “partial” indicates that multiple sequences of the v1 match one sequence of the v0, numbers indicate how may genes from one set match one gene from the other set. Cardinality between 8X and 12Xv1: Comment about the accuracy of the gene prediction inferred from the 8X to 12X comparison; “merge” indicates that multiple sequences of the 8X assembly match one sequence of the 12Xv1 (unless noted otherwise the 12X assembly gene is correct), “To split” indicates that the 12X gene is incorrect and needs to be split (if there are more than 2 genes, those that need to be grouped are indicated by order in the column “Identical genes in 8X or other EST”), “redundant” indicates multiple12X genes matching a single 8X gene on the same position, XX indicates no match between 12X a [file 1756-0500-5-213-S1.tiff]
