# Supplementary material for: Comparative analysis of grapevine whole-genome gene predictions, functional annotation, categorization and integration of the predicted gene sequences
Source: BMC Res Notes. 2012 May 3;5:213. doi: 10.1186/1756-0500-5-213 (PMC3419625; doi:10.1186/1756-0500-5-213)
Supplement: Additional file 4 — Analyses workflow for determining cardinality between 8X and 12Xv1 assembly genes. Straight line: representation of the genes from the 12Xv1 assembly. Wavy line: representation of the genes from the 8X assembly. Dotted line: genetic sequence. [file 1756-0500-5-213-S4.docx]

**Additional file 4.** List of networks available in VitisNet. VVID: VitisNet identification number; gen: number of genes in network; pro: number of proteins in network; met: number of metabolites in network. Italic: new networks.

| **1** | **Metabolism** |  |  |  |  |  |  |  |  |
| --- | --- | --- | --- | --- | --- | --- | --- | --- | --- |
| **VVID** | **Network name** | **gen** | **pro** | **met** | **VVID** | **Network name** | **gen** | **pro** | **met** |
| **1.1** | **Carbohydrate metabolism** |  |  |  |  |  |  |  |  |
| 10010 | Glycolysis | 223 | 29 | 28 | 10530 | Aminosugars met. | 108 | 8 | 10 |
| 10020 | Citrate cycle | 79 | 18 | 21 | 10520 | Nucleotide sugars met. | 72 | 16 | 18 |
| 10030 | Pentose phosphate | 89 | 17 | 21 | 10620 | Pyruvate met. | 230 | 28 | 19 |
| 10040 | Pentose/glucuron. interconv. | 62 | 11 | 14 | 10630 | Glyoxyl., dicarboxyl. met. | 88 | 18 | 19 |
| 10051 | Fructose and mannose met. | 117 | 20 | 20 | 10640 | Propanoate met. | 71 | 11 | 12 |
| 10052 | Galactose met. | 176 | 16 | 27 | 10650 | Butanoate met. | 88 | 18 | 19 |
| 10053 | Ascorbate and aldarate met. | 53 | 9 | 9 | 10562 | Inositol phosphate met. | 70 | 18 | 20 |
| 10500 | Starch and sucrose met. | 373 | 47 | 34 |  |  |  |  |  |
| **1.2** | **Energy metabolism** |  |  |  |  |  |  |  |  |
| 10190 | Oxidative phosphorylation | 383 | 105 | 7 | 10720 | Red. carboxylate cycle | 44 | 10 | 14 |
| 10195 | Photosynthesis | 223 | 52 |  | 10680 | Methane met. | 133 | 10 | 11 |
| 10196 | Photosynthesis antenna prot. | 38 | 11 |  | 10910 | Nitrogen met. | 117 | 23 | 19 |
| 10710 | Carbon fixation | 182 | 21 | 20 | 10920 | Sulfur met. | 48 | 13 | 12 |
| **1.3** | **Lipid Metabolism** |  |  |  |  |  |  |  |  |
| 10061 | Fatty acid biosynthesis | 86 | 13 | 36 | 10561 | Glycerolipid met. | 159 | 19 | 18 |
| 10062 | Fatty acid elong. in mitoch. | 29 | 7 | 29 | 10564 | Glycerophospholipid met. | 150 | 34 | 32 |
| 10071 | Fatty acid met. | 111 | 30 | 40 | 10565 | Ether lipid met. | 53 | 13 | 9 |
| 10072 | Synth./deg. of ketone bodies | 20 | 3 | 4 | 10600 | Sphingolipid met. | 77 | 13 | 15 |
| 10100 | Biosynthesis of steroids | 150 | 49 | 76 | 10592 | Alpha-linolenic acid met. | 106 | 21 | 29 |
| 10140 | C21-Steroid hormone met. | 18 | 6 | 14 | 11040 | Biosynth. unsat. fatty acids | 54 | 20 | 27 |
| **1.4** | **Nucleotide metabolism** |  |  |  |  |  |  |  |  |
| 10230 | Purine met. | 154 | 49 | 62 | 10240 | Pyrimidine met. | 113 | 39 | 46 |
| **1.5** | **Amino Acid metabolism** |  |  |  |  |  |  |  |  |
| 10251 | Glutamate metabolism | 97 | 28 | 25 | 10330 | Arginine and proline met. | 65 | 17 | 23 |
| 10252 | Alanine and aspartate met. | 117 | 24 | 24 | 10340 | Histidine metabolism | 65 | 18 | 19 |
| 10260 | Gly. ser. and threonine met. | 116 | 31 | 38 | 10350 | Tyrosine metabolism | 165 | 27 | 39 |
| 10271 | Methionine metabolism | 135 | 32 | 47 | 10360 | Phenylalanine metabolism | 224 | 17 | 14 |
| 10272 | Cysteine metabolism | 84 | 17 | 25 | 10380 | Tryptophan metabolism | 19 | 6 | 7 |
| 10280 | Val. leu. and ile degradation | 92 | 20 | 34 | 10400 | Phe. tyr. and trp biosynthesis | 159 | 30 | 35 |
| 10290 | Val. leu. and ile biosynthesis | 73 | 14 | 26 | 10220 | Urea cyc., met. amino grps | 134 | 33 | 41 |
| 10300 | Lysine biosynthesis | 85 | 52 | 22 |  |  |  |  |  |
| **1.6** | **Other amino acids metabolism** | | | | | | | | |
| 10410 | Beta-alanine met. | 59 | 13 | 13 | 10460 | Cyanoamino acid met. | 39 | 8 | 16 |
| 10450 | Selenoamino acid met. | 82 | 15 | 17 | 10480 | Glutathione met. | 159 | 37 | 16 |
| **1.7** | **Glycan biosynthesis and metabolism** | | | | | |  |  |  |
| 10510 | N-Glycan biosynthesis | 56 | 23 | 22 | 10563 | GPI-anchor biosynth. | 22 | 12 | 14 |
| 10511 | N-Glycan degradation | 780 | 8 |  | 10602 | Glycosphingolipid biosynth. | 17 | 7 | 16 |
| 10540 | Lipopolysacch. biosynth. | 11 | 10 | 13 | 11030 | Glycan structures-biosynth.1 | 101 | 26 | 48 |
| 10550 | Peptidoglycan biosynthesis | 20 | 3 | 10 |  |  |  |  |  |
| **1.8** | **Cofactors and vitamin metabolism** | | | | | | | |  |
| 10730 | Thiamine metabolism | 22 | 12 | 20 | 10780 | Biotin metabolism | 8 | 6 | 8 |
| 10740 | Riboflavin metabolism | 70 | 13 | 15 | 10790 | Folate biosynthesis | 40 | 17 | 24 |
| 10750 | Vitamin B6 metabolism | 19 | 7 | 13 | 10670 | One carbon pool by folate | 49 | 15 | 9 |
| 10760 | Nicotinate/nicotinamide met. | 28 | 12 | 13 | 10860 | Porphyrin/chlorophyll met. | 75 | 32 | 39 |
| 10770 | Pantothenate/CoA biosynth. | 56 | 15 | 19 | 10130 | Ubiquinone biosynthesis | 36 | 17 | 25 |
| **1.9** | **Biosynthesis of secondary metabolites** | | | | | |  |  |  |
| 10900 | Terpenoid biosynthesis | 192 | 18 | 24 | 10942 | Anthocyanin biosynthesis | 72 | 8 | 18 |
| 10902 | Monoterpenoid biosynthesis | 217 | 29 | 42 | 10943 | Isoflavonoid biosynthesis | 68 | 7 | 17 |
| 10904 | Diterpenoid biosynthesis | 78 | 19 | 37 | 10950 | Alkaloid biosynthesis I | 65 | 17 | 23 |
| 10908 | Zeatin biosynthesis | 61 | 11 | 20 | 10311 | Penicillin/cephalosp. bioS. | 13 | 5 | 5 |
| 10906 | Carotenoid biosynthesis | 49 | 22 | 33 | 11002 | Auxin biosynthesis | 99 | 24 | 12 |
| 10905 | Brassinosteroid biosynthesis | 21 | 7 | 24 | 11012 | Iba metabolism | 21 | 11 | 5 |
| 10940 | Phenylpropanoid biosynth. | 243 | 25 | 46 | *11013* | *ABA biosynthesis* | *18* | *8* | *11* |
| 10941 | Flavonoid biosynthesis | 190 | 25 | 52 | *10966* | *Glucosinolate biosynthesis* | *40* | *10* | *27* |
| **1.10** | **Other** |  |  |  |  |  |  |  |  |
| 11000 | Single reactions | 190 | 15 | 38 |  |  |  |  |  |
| **2** | **Genetic information processing networks** |  |  |  |  |  |  |  |  |
| **VVID** | **Network name** | **gen** | **pro** | **met** | **VVID** | **Network name** | **gen** | **pro** | **met** |
| **2.1** | **Transcription** |  |  |  |  |  |  |  |  |
| 23020 | RNA polymerase | 96 | 34 |  | *23040* | *Spliceosome* | *280* | *139* |  |
| 23022 | Basal transcription factors | 67 | 23 |  |  |  |  |  |  |
| **2.2** | **Translation** |  |  |  |  |  |  |  |  |
| 23010 | Ribosome | 612 | 131 |  | *23015* | *mRNA surveillance pathway* | *135* | *42* |  |
| 20970 | Aminoacyl-tRNA biosynth. | 122 | 22 | 64 | *23008* | *Ribosome biogenesis in Euk.* | *141* | *59* |  |
| *23013* | *RNA transport* | *260* | *92* |  |  |  |  |  |  |
| **2.3** | **Folding, sorting degradation** | | | |  |  |  |  |  |
| 23060 | Protein export | 64 | 30 |  | 23050 | Proteasome | 75 | 47 |  |
| *24141* | *Protein processing in ER* | *279* | *68* |  | 24120 | Ubiquitin med. proteolysis | 204 | 68 | 3 |
| 24130 | SNARE int. in ves. transport | 69 | 22 |  |  |  |  |  |  |
| **2.4** | **Replication and repair** |  |  |  |  |  |  |  |  |
| 23030 | DNA replication | 82 | 38 |  | 23440 | Homologous recombination | 51 | 19 |  |
| 23410 | Base excision repair | 32 | 21 |  | 23450 | Non homologous end joining | 19 | 8 |  |
| 23420 | Nucleotide excision repair | 72 | 36 |  | *24122* | *Sulfur relay system* | *15* | *7* | *2* |
| 23430 | Mismatch repair | 49 | 19 |  | *23018* | *RNA degradation* | *115* | *48* |  |
| **3** | **Environmental information processing** | | | | |  |  |  |  |
| **VVID** | **Network name** | **gen** | **pro** | **met** | **VVID** | **Network name** | **gen** | **pro** | **met** |
| **3.1** | **Signal transduction** |  |  |  |  |  |  |  |  |
| 34020 | Calcium signaling pathway | 152 | 32 | 22 | 34150 | mTOR signaling pathway | 32 | 17 |  |
| 34070 | Phosphatidylinositol sig. sys. | 83 | 14 | 17 |  |  |  |  |  |
| **3.2** | **Hormone signaling** |  |  |  |  |  |  |  |  |
| 30001 | ABA signaling | 168 | 101 | 11 | 30008 | Ethylene signaling | 275 | 247 | 3 |
| 30003 | Auxin signaling | 311 | 149 | 1 | 30010 | Gibberellin signaling | 43 | 28 | 1 |
| 30005 | Brassinosteroids signaling | 63 | 24 | 2 | 30011 | Jasmonate signaling | 98 | 71 | 4 |
| 30007 | Cytokinin signaling | 80 | 74 | 1 |  |  |  |  |  |
| **3.3** | **Plant-specific signaling** |  |  |  |  |  |  |  |  |
| 34710 | Circadian rhythm | 79 | 63 |  | *34627* | *R prot. from Plant-path. int.* | *472* | *11* |  |
| *34626* | *Plant-pathogen interaction* | *360* | *122* | 7 | 30009 | Flower development | 204 | 188 | 1 |
| **4** | **Cellular processes** |  |  |  |  |  |  |  |  |
| **VVID** | **Network name** | **gen** | **pro** | **met** | **VVID** | **Network name** | **gen** | **Pro** | **met** |
| **4.1** | **Transport and catabolism** |  |  |  |  |  |  |  |  |
| 44140 | Regulation of autophagy | 30 | 16 | 4 | *44146* | *Peroxisome* | *95* | *47* | *1* |
| *44145* | *Phagosome* | *142* | *29* |  |  |  |  |  |  |
| **4.2** | **Cell motility** |  |  |  |  |  |  |  |  |
| 44810 | Reg. of actin cytoskeleton | 396 | 117 | 1 |  |  |  |  |  |
| **4.3** | **Cell growth and death** |  |  |  |  |  |  |  |  |
| 44110 | Cell cycle | 358 | 238 |  | 40006 | Cell wall | 518 | 62 | 11 |
| **5** | **Transport** |  |  |  |  |  |  |  |  |
| **VVID** | **Network name** | **gen** | **pro** | **met** | **VVID** | **Network name** | **gen** | **pro** | **met** |
| **5.1** | **Membrane transport** |  |  |  |  |  |  |  |  |
| 52010 | ABC transporters | 284 | 93 |  |  |  |  |  |  |
| **5.2** | **Hormone transport** |  |  |  |  |  |  |  |  |
| 50004 | Auxin transport | 58 | 27 | 1 |  |  |  |  |  |
| **5.3** | **Transport system** |  |  |  |  |  |  |  |  |
| 50110 | Protein coat | 229 | 105 |  | 50112 | Nuclear pore complex | 90 | 28 |  |
| 50111 | Tethering factors | 117 | 70 |  | 50113 | Thylakoid targeting pathway | 71 | 15 |  |
| **5.4** | **Transporter catalog** |  |  |  |  |  |  |  |  |
| 50101 | Channels and pores | 451 | 146 |  | 50124 | Porters cat 30 to 64 | 170 | 70 |  |
| 50104 | Group translocators | 42 | 10 |  | 50125 | Porters cat 66 to 94 | 230 | 55 |  |
| 50105 | Transport electron carriers | 109 | 38 |  | 50131 | Prim. active transp. A2-A4 | 223 | 53 |  |
| 50108 | Accessory fact. inv. in transp | 172 | 16 |  | 50132 | Prim. active transp. A5-A8 | 205 | 84 |  |
| 50109 | Incompletely characterized transport systems | 383 | 98 |  | 50133 | Prim. active transp. A9-A18 | 262 | 99 |  |
| 50121 | Porters cat 1 to 6 | 197 | 86 |  | 50134 | Prim. active transp. cat D1 | 175 | 46 |  |
| 50122 | Porters cat 7 to 17 | 259 | 50 |  | 50135 | Prim. active transp. D3-E2 | 156 | 44 |  |
| 50123 | Porters cat 18 to 29 | 225 | 46 |  |  |  |  |  |  |
| **6** | **Transcription factors** |  |  |  |  |  |  |  |  |
| **VVID** | **Network name** | **gen** | **pro** | **met** | **VVID** | **Network name** | **gen** | **pro** | **met** |
| 60001 | ABI3VP1 | 25 | 25 |  | 60049 | PLATZ | 12 | 12 |  |
| 60002 | Alfin-like | 8 | 8 |  | 60050 | PseudoARR-B | 9 | 9 |  |
| 60003 | AP2 EREBP | 158 | 158 |  | 60051 | RB | 2 | 2 |  |
| 60004 | ARF | 29 | 29 |  | 60052 | RWP-RK | 12 | 12 |  |
| 60005 | ARID | 12 | 12 |  | 60053 | S1Fa-like | 3 | 3 |  |
| 60006 | ARR-B | 15 | 15 |  | 60054 | SAP | 1 | 1 |  |
| 60007 | AS2 | 52 | 51 |  | 60055 | SBP | 23 | 23 |  |
| 60008 | AUXIAA | 31 | 31 |  | 60056 | SET PCG | 64 | 64 |  |
| 60009 | BBR | 6 | 6 |  | 60057 | Sigma70-like | 6 | 6 |  |
| 60010 | BES1 | 11 | 11 |  | 60058 | SNF2 | 47 | 47 |  |
| 60011 | BHLH | 163 | 163 |  | 60059 | SRS | 5 | 5 |  |
| 60012 | BZIP | 73 | 73 |  | 60060 | TAZ | 8 | 7 |  |
| 60013 | BHSH | 1 | 1 |  | 60061 | TCP | 21 | 21 |  |
| 60014 | C2C2-CO | 14 | 14 |  | 60062 | Trihelix | 37 | 37 |  |
| 60015 | C2C2-DOF | 25 | 25 |  | 60063 | TUB | 17 | 17 |  |
| 60016 | C2C2-GATA | 25 | 25 |  | 60064 | ULT | 1 | 1 |  |
| 60017 | C2H2 | 138 | 138 |  | 60065 | VOZ | 2 | 2 |  |
| 60018 | C3H | 93 | 93 |  | 60066 | WRKY | 69 | 69 |  |
| 60019 | C2C2-YABBY | 7 | 7 |  | 60067 | zf-MYND | 4 | 4 |  |
| 60020 | CAMTA | 8 | 8 |  | 60068 | zf-HD | 18 | 18 |  |
| 60021 | CCAAT | 35 | 35 |  | 60069 | ZIM | 14 | 14 |  |
| 60022 | CPP | 7 | 7 |  | 60070 | Orphans CCT | 8 | 8 |  |
| 60023 | CSD | 4 | 4 |  | 60071 | Orphans FAR-RED | 71 | 71 |  |
| 60024 | DBP | 7 | 7 |  | 60072 | Orphans Response reg | 16 | 16 |  |
| 60025 | DDT | 10 | 10 |  | 60073 | Orphans zf-b box | 15 | 15 |  |
| 60026 | E2F-DP | 8 | 8 |  | 60074 | Orphans zf-SWIM | 8 | 8 |  |
| 60027 | EIL | 5 | 5 |  | 60075 | Other BSD | 12 | 12 |  |
| 60028 | FHA | 23 | 23 |  | 60076 | Other GTF | 7 | 7 |  |
| 60029 | G2-like | 43 | 43 |  | 60077 | Other zf-AN1 | 15 | 15 |  |
| 60030 | GeBP | 8 | 8 |  | 60078 | Other zf-C3HC4 | 279 | 279 |  |
| 60031 | GIF | 4 | 4 |  | 60079 | Other zf-DHHC | 25 | 25 |  |
| 60032 | GRAS | 53 | 53 |  | 60080 | Other zf | 39 | 39 |  |
| 60033 | GRF | 14 | 14 |  | *60081* | *COAP15* | *2* | *2* |  |
| 60034 | HB | 102 | 102 |  | *60082* | *GNAT* | *36* | *36* |  |
| 60035 | HMG | 18 | 18 |  | *60083* | *IWS1* | *1* | *1* |  |
| 60036 | HRT | 1 | 1 |  | *60084* | *MED* | *2* | *2* |  |
| 60037 | HSF | 22 | 22 |  | *60085* | *MTERF* | *39* | *39* |  |
| 60038 | Jumonji | 29 | 29 |  | *60086* | *OFP* | *17* | *17* |  |
| 60039 | LFY | 1 | 1 |  | *60087* | *Orphans KIP1 SANTA* | *6* | *6* |  |
| 60040 | LIM | 12 | 12 |  | *60088* | *Orphans WRC* | *1* | *1* |  |
| 60041 | LUG | 9 | 9 |  | *60089* | *Other Nozzle* | *1* | *1* |  |
| 60042 | MADS | 81 | 81 |  | *60090* | *RCD1* | *2* | *2* |  |
| 60043 | MBF1 | 6 | 6 |  | *60091* | *SOH1* | *1* | *1* |  |
| 60044 | MYB | 179 | 179 |  | *60092* | *SWIB* | *16* | *16* |  |
| 60045 | MYBrelated | 71 | 71 |  | *60093* | *TRAF* | *34* | *34* |  |
| 60046 | NAC | 86 | 86 |  | *60094* | *SWI SNF SWI3* | *2* | *2* |  |
| 60047 | PBF-2-like | 2 | 2 |  | *60095* | *Orphans PAH* | *4* | *4* |  |
| 60048 | PHD | 81 | 81 |  |  |  |  |  |  |
